# Normal Human Lung Epithelial Cells Inhibit Transforming Growth Factor-β Induced Myofibroblast Differentiation via Prostaglandin E_2_

**DOI:** 10.1371/journal.pone.0135266

**Published:** 2015-08-06

**Authors:** Amali P. Epa, Thomas H. Thatcher, Stephen J. Pollock, Lindsay A. Wahl, Elizabeth Lyda, R. M. Kottmann, Richard P. Phipps, Patricia J. Sime

**Affiliations:** 1 Department of Pathology, University of Rochester School of Medicine and Dentistry, 601 Elmwood Avenue, Rochester, NY 14642, United States of America; 2 Lung Biology and Disease Program, University of Rochester School of Medicine and Dentistry, 601 Elmwood Avenue, Rochester, NY 14642, United States of America; 3 Division of Pulmonary and Critical Care Medicine, Department of Medicine, University of Rochester, 601 Elmwood Avenue, Rochester, NY 14642, United States of America; 4 Department of Microbiology and Immunology, University of Rochester School of Medicine and Dentistry, 601 Elmwood Avenue, Rochester, NY 14642, United States of America; 5 Department of Environmental Medicine, University of Rochester School of Medicine and Dentistry, 601 Elmwood Avenue, Rochester NY, 14642, United States of America; University of Alabama at Birmingham, UNITED STATES

## Abstract

**Introduction:**

Idiopathic pulmonary fibrosis (IPF) is a chronic progressive disease with very few effective treatments. The key effector cells in fibrosis are believed to be fibroblasts, which differentiate to a contractile myofibroblast phenotype with enhanced capacity to proliferate and produce extracellular matrix. The role of the lung epithelium in fibrosis is unclear. While there is evidence that the epithelium is disrupted in IPF, it is not known whether this is a cause or a result of the fibroblast pathology. We hypothesized that healthy epithelial cells are required to maintain normal lung homeostasis and can inhibit the activation and differentiation of lung fibroblasts to the myofibroblast phenotype. To investigate this hypothesis, we employed a novel co-culture model with primary human lung epithelial cells and fibroblasts to investigate whether epithelial cells inhibit myofibroblast differentiation.

**Measurements and Main Results:**

In the presence of transforming growth factor (TGF)-β, fibroblasts co-cultured with epithelial cells expressed significantly less α-smooth muscle actin and collagen and showed marked reduction in cell migration, collagen gel contraction, and cell proliferation compared to fibroblasts grown without epithelial cells. Epithelial cells from non-matching tissue origins were capable of inhibiting TGF-β induced myofibroblast differentiation in lung, keloid and Graves’ orbital fibroblasts. TGF-β promoted production of prostaglandin (PG) E_2_ in lung epithelial cells, and a PGE_2_ neutralizing antibody blocked the protective effect of epithelial cell co-culture.

**Conclusions:**

We provide the first direct experimental evidence that lung epithelial cells inhibit TGF-β induced myofibroblast differentiation and pro-fibrotic phenotypes in fibroblasts. This effect is not restricted by tissue origin, and is mediated, at least in part, by PGE_2_. Our data support the hypothesis that the epithelium plays a crucial role in maintaining lung homeostasis, and that damaged and/ or dysfunctional epithelium contributes to the development of fibrosis.

## Introduction

Fibrosis refers to the process of excessive accumulation of scar tissue, and occurs in a variety of chronic diseases affecting organs as diverse as the lung, kidney, eye, heart and skin. Abnormal activation and proliferation of fibroblasts is accompanied by excess production of extracellular matrix proteins and an imbalance in matrix turnover are hallmarks of fibrotic disorders [1, 2]. Tissue fibrosis is responsible for significant morbidity and mortality related to organ failure and occurs when there is dysregulation of normal wound healing.

Idiopathic pulmonary fibrosis (IPF) is a severe form of pulmonary fibrosis, in which the underlying pathophysiology remains poorly understood [3, 4]. Unlike other interstitial lung diseases, such as silicosis, where the initial injury/insult is known, the causes of IPF remain elusive. An emerging concept is that normal interactions between epithelium and the mesenchyme play an important role in maintaining lung homeostasis, and that damaged lung epithelium contributes to pulmonary fibrosis [5–8]. For example, lung epithelial cells were shown to be an important site of production of pro-fibrotic factors including TGF-β, TNF-α and PDGF [9–12]. Furthermore, fibroblastic foci are associated with damaged epithelial cells [8], and a recent study showed that injury directed to type II alveolar epithelial cells increases collagen accumulation in the lung in a mouse model [13]. However, it remains unclear as to whether epithelial damage is a cause of fibrosis or is a result of the presence of excess myofibroblasts and fibroblastic foci [14]. The role played by healthy lung epithelium in maintaining homeostasis remains largely unexplored.

Prostaglandin E_2_ (PGE_2_) is the major arachidonic acid metabolite produced by alveolar epithelial cells (AECs) in humans. Patients with IPF were found to have significantly reduced amounts of PGE_2_ in the epithelial lining fluids [15]. Early studies using rat and mouse alveolar epithelial cells showed that epithelial cells inhibit fibroblast proliferation by directly secreting PGE_2_ or indirectly inducing fibroblast PGE_2_ secretion. [16–18] Although multiple reports have shown that addition of exogenous PGE_2_ inhibits pro-fibrotic functions of myofibroblasts *in vitro* [19–21], no one has yet investigated whether human lung epithelial PGE_2_ might play a role in maintaining normal lung homeostasis by inhibiting the effects of pro-fibrotic insults.

Here, we provide the first direct experimental evidence that normal human lung epithelial cells can prevent the development of a pro-fibrotic phenotype in human lung fibroblasts, both from normal subjects and patients with IPF. This effect is mediated by PGE_2_, and is not confined to cells of lung origin, as epithelial cells from multiple tissues can inhibit myofibroblast differentiation. Our data reinforces the concept that fibrosing diseases are indeed involve disordered epithelial-fibroblast crosstalk, and encourages the importance of additional investigations of cell-cell communication in lung disease.

## Materials and Methods

### Cell Culture Studies

All patient samples were obtained with written informed consent under the approval of the University of Rochester Institutional Review Board. Primary human alveolar epithelial cells were isolated from subjects undergoing lung biopsy for suspected new or metastatic lung cancer. AECs were harvested from tissue distal to the nodules as previously described [22], viability and purity were assessed by trypan blue method, and modified papanicolaou staining. Purified cells were grown on rat tail collagen coated tissue culture plates in Dulbecco’s modified Eagle’s medium (DMEM) (Gibco) supplemented with 10% fetal bovine serum. After two days in culture serum levels were reduced to 5%, cells continue to express surfactant protein-C (Pro-SPC) as assessed by Immunocytochemistry. Primary human lung fibroblasts (HLFs) were derived and grown as previously described [23, 24]. Primary human small airway epithelial cells (SAEC) were purchased from Lonza (Allendale, NJ) and maintained in Small Airway Epithelial Cell Growth Medium (SAGM; basal medium plus growth supplements, Lonza). Cells were used between passage 4 and 10. Primary keloid fibroblasts and Graves’ orbital fibroblasts [25] were grown in Dulbecco’s modified Eagle’s medium (DMEM) (Gibco) supplemented with 10% fetal bovine serum, 100 U/ml penicillin/streptomycin and 2mM L-glutamine. Primary human neonatal epidermal keratinocytes (HEKn) were grown in SAGM.

### Reagents

Recombinant human TGF-β1 was purchased from R&D Systems (Minneapolis, MN). Prostaglandin E_2_ (PGE_2_), anti-COX-2 antibody, COX-2 inhibitor SC-58125, and prostaglandin E_2_ monoclonal antibody (2B5) were purchased from Cayman Chemicals (Ann Arbor, MI). Lyophilized porcine pancreatic elastase was purchased from Worthington Biochemical Corporation (Lakewood, NJ). CD-45 Dynabeads were purchased from Invitrogen (Carlsbad, CA). OptiPrep was purchased from Sigma-Aldrich (St. Louis, MO). Rat-tail collagen was purchased from Roche (Germany).

### Fibroblast-SAEC Co-culture

**Fig 1:**
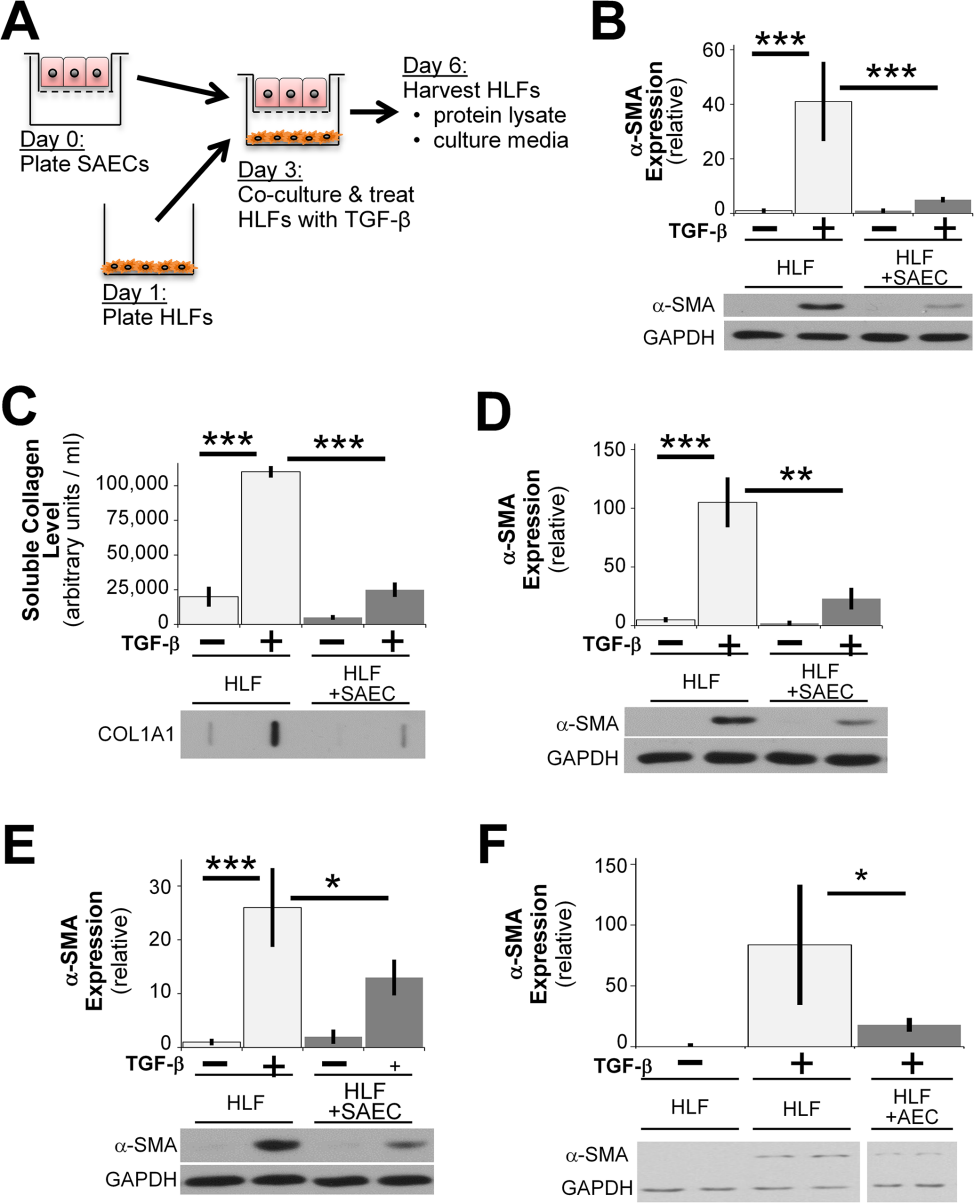
SAECs inhibit TGF-β induced pro-fibrotic protein expression in human lung fibroblasts. (A) A schematic of co-culture system of HLFs and SAECs. HLFs and SAECs were grown separately on lower wells and upper inserts, respectively, of a Transwell co-culture system. HLFs were washed with PBS, co-cultured with SAECs with or without TGF-β for 72 hours (unless otherwise indicated). (B) HLFs were treated with 5ng/ml TGF- β in the presence or absence SAECs and α-SMA protein expression was analyzed by western blot and densitometric analysis. (C) Soluble collagen in culture medium from co-cultures was measured by slot blot with densitometric analysis. (D, E) HLFs were co-cultured with SAECs from two additional (different donors). Blots are representative of at least three independent experiments. (F) Alveolar epithelial cells were co-cultured with HLFs, and HLF expression of α-SMA was determined by western blot. Note that in Fig 1F, the indicated samples were resolved on the same gel, and intervening irrelevant lanes are not shown. Densitometry of n = 3 replicates per cell strain, normalized to untreated control. Data shown are mean ± SD. *** = p<0.001 ** = p<0.01 and * = p<0.05 by ANOVA.

On day 0, epithelial cells were plated on the apical side of 0.4 μm cell culture inserts (Transwell Permeable Support, Corning, NY) in SAGM. On day 1, fibroblasts were plated in 12-well plates in supplemented MEM. On day 3, the fibroblasts were washed with PBS and changed to SAGM, and culture inserts with SAECs were added to each well of fibroblasts. The cells maintained as co-cultures with indicated treatments for 72 hours unless otherwise indicated (Fig 1A).

### Fibroblast-AEC Co-culture

On day 0, primary AECs were plated on the apical side of rat tail collagen coated 0.4 μm cell culture inserts in DMEM supplemented with 10% FBS. On day 1, fibroblasts were plated in 12-well plates in supplemented MEM. On day 3, the fibroblasts were washed with PBS and co-cultured with AECs in DMEM-5% FBS with indicated treatments for 72 hours.

### Western Blot

Whole cell lysates were prepared using NP-40 lysis buffer with protease inhibitors (Sigma-Aldrich) and analyzed as previously described [26]. Protein expression was detected using α-SMA (Sigma-Aldrich, St. Louis, MO), GAPDH (Abcam, Cambridge, MA) and COX-2 primary antibodies with horseradish peroxidase (HRP)-conjugated secondary antibodies (Jackson ImmunoResearch, West Grove, PA) and visualized by enhanced chemiluminescence.

### Collagen Slot Blot

7μl aliquots of cell culture medium were applied to Immobilon-P PVDF membranes under gentle vacuum using a slot blot manifold (Harvard Apparatus). Soluble collagen was detected using COL1A1 (L-19) antibody (Santa Cruz Biotechnology) followed by HRP-conjugated secondary antibodies and visualized by enhanced chemiluminescence.

### Collagen Gel Contraction Assay

HLFs were seeded within collagen gels as previously described [27], except that gels were floated in SAGM containing indicated experimental treatments. For co-culture experiments, SAECs cultured in Transwell inserts were added to wells containing fibroblast-seeded collagen gels.

### Cell Migration Assays

HLF migration assay was performed as previously described [27], except when co-cultured, HLFs were incubated with SAECs grown in Transwell inserts as described above.

### Fibroblast Proliferation

HLFs were plated onto 96-well tissue culture plates. 24 hours later cells were washed with PBS, and treated in fresh SAGM or SAEC-conditioned medium with or without TGF-β for 24 hours. [^3^H]-thymidine (1 uCi/μl) was added to cells for an additional 18 hours. [^3^H]-thymidine incorporation was determined as previously described [28] [29].

### PGE_2_ Quantification

PGE_2_ in cell culture medium were quantified using PGE_2_ Express EIA Kit according to manufacturer’s instructions (Cayman Chemicals, Ann Arbor, MI).

### PGE_2_ Neutralization

Conditioned medium was incubated with 10 μg/ml of 2B5 anti-PGE_2_ antibody for 30 minutes at room temperature prior to treating the HLFs [30].

### Statistics

Statistical analysis was conducted using either one-way ANOVA with Tukey post-test or Student’s t-test using Prism software version 5 (Graphpad, La Jolla, CA). P values are listed in the figure legends. Results were considered significant when p<0.05. All raw data for the figures shown in this manuscript is contained in an online data supplement.

## Results

### Human lung epithelial cells inhibit TGF-β induced α-SMA expression in human lung fibroblasts

To determine whether lung epithelial cells are capable of exerting anti-fibrotic effects on fibroblasts, we designed a co-culture system in which normal primary human lung fibroblasts (HLFs) are co-cultured with primary human small airway epithelial cells (SAECs) separated by permeable culture inserts (Fig 1A). HLFs were treated with or without TGF-β1 (5ng/ml) in the presence or absence of epithelial cells for 72 hours. Fibroblast lysates were assessed for expression of α-smooth muscle actin (SMA), a marker of myofibroblast differentiation, by western blot. HLFs stimulated with TGF-β1 differentiated to myofibroblasts and expressed α-SMA, as we and others have reported [26, 31, 32]. However, when co-cultured with SAECs, myofibroblast differentiation after TGF-β1 stimulation was almost completely inhibited (Fig 1B). In addition to reducing α-SMA expression, co-culture with SAECs significantly reduced soluble collagen expression by TGF-β treated HLFs compared to HLFs that were cultured alone (Fig 1C). The ability of SAECs to inhibit myofibroblast differentiation was a consistent property of SAEC strains from multiple donors (Fig 1D and 1E). Because it is currently unknown which type(s) of lung epithelial cells may be most important in IPF, we also examined the effect of freshly isolated alveolar epithelial cells (AECs). AECs also potently inhibited TGF-β induced myofibroblast differentiation of human lung fibroblasts (Fig 1F).

### SAECs inhibit TGF-β induced α-SMA expression in both normal and fibrotic human lung fibroblasts

**Fig 2:**
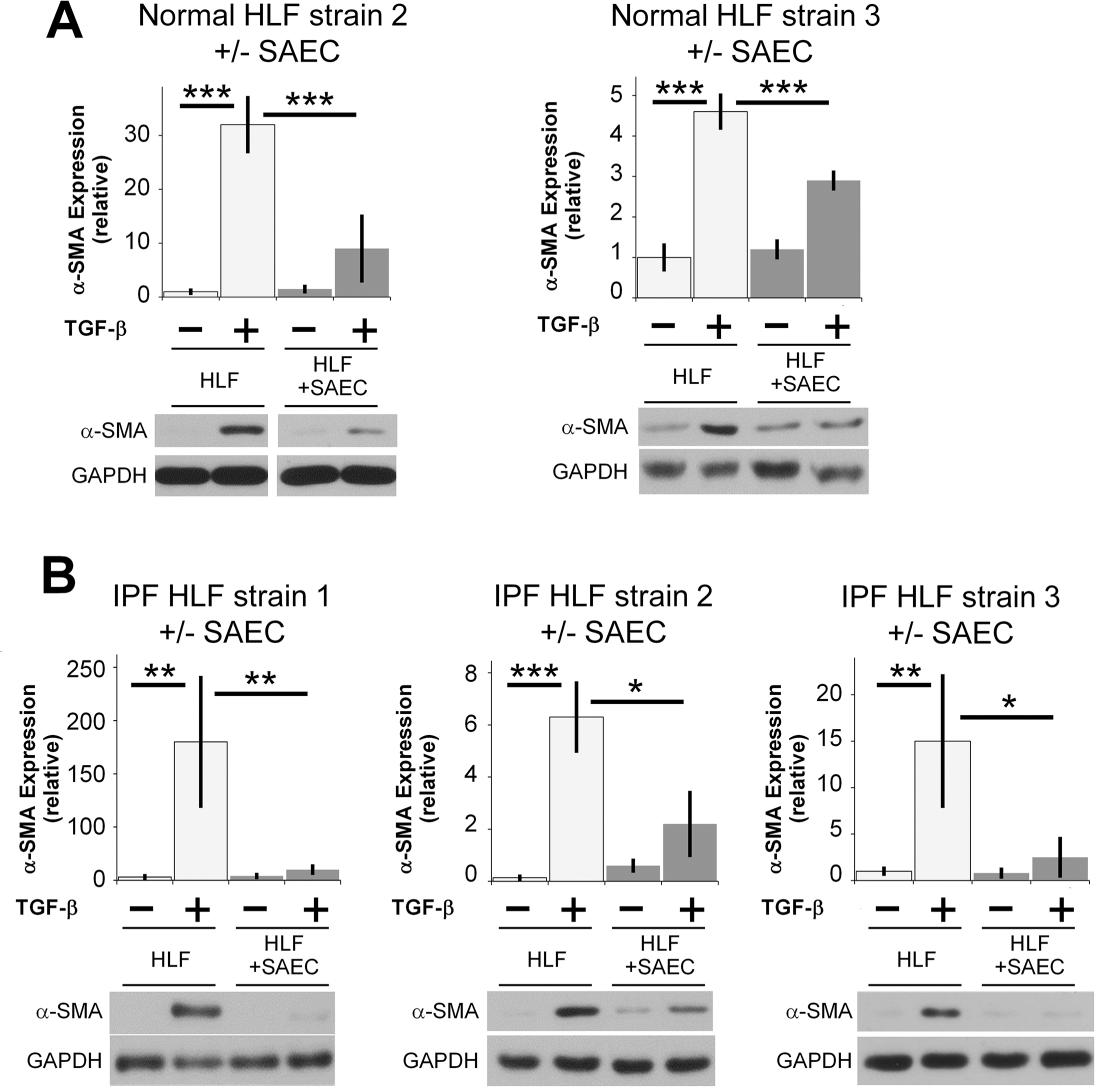
SAECs inhibit TGF-β induced α-SMA protein expression in multiple normal and fibrotic human lung fibroblast strains. (A) Two additional normal, and (B) three additional IPF fibroblast strains from different donors were treated with or without 5ng/ml TGF-β and co-cultured with SAECs for 72 hours. α-SMA protein expression was analyzed by western blot. Representative blots are shown. Note that in Fig 2A, the indicated samples were resolved on the same gel, and intervening irrelevant lanes are not shown. Densitometry of n = 3 replicates per cell strain, normalized to untreated control. Data shown are mean ± SD. *** = p<0.001 ** = p<0.01 and * = p<0.05 by ANOVA.

The ability of SAECs to suppress myofibroblast differentiation was also a consistent property of multiple lung fibroblast strains. We evaluated primary HLFs from 2 additional non-fibrotic donors and from 3 IPF patients. Interestingly, SAECs were potently effective at inhibiting TGF-β stimulated myofibroblast differentiation in both normal and fibrotic HLFs (Fig 2A and 2B).

### Epithelial cells inhibit TGF-β induced α-SMA expression in fibroblasts irrespective of their tissue origin

**Fig 3:**
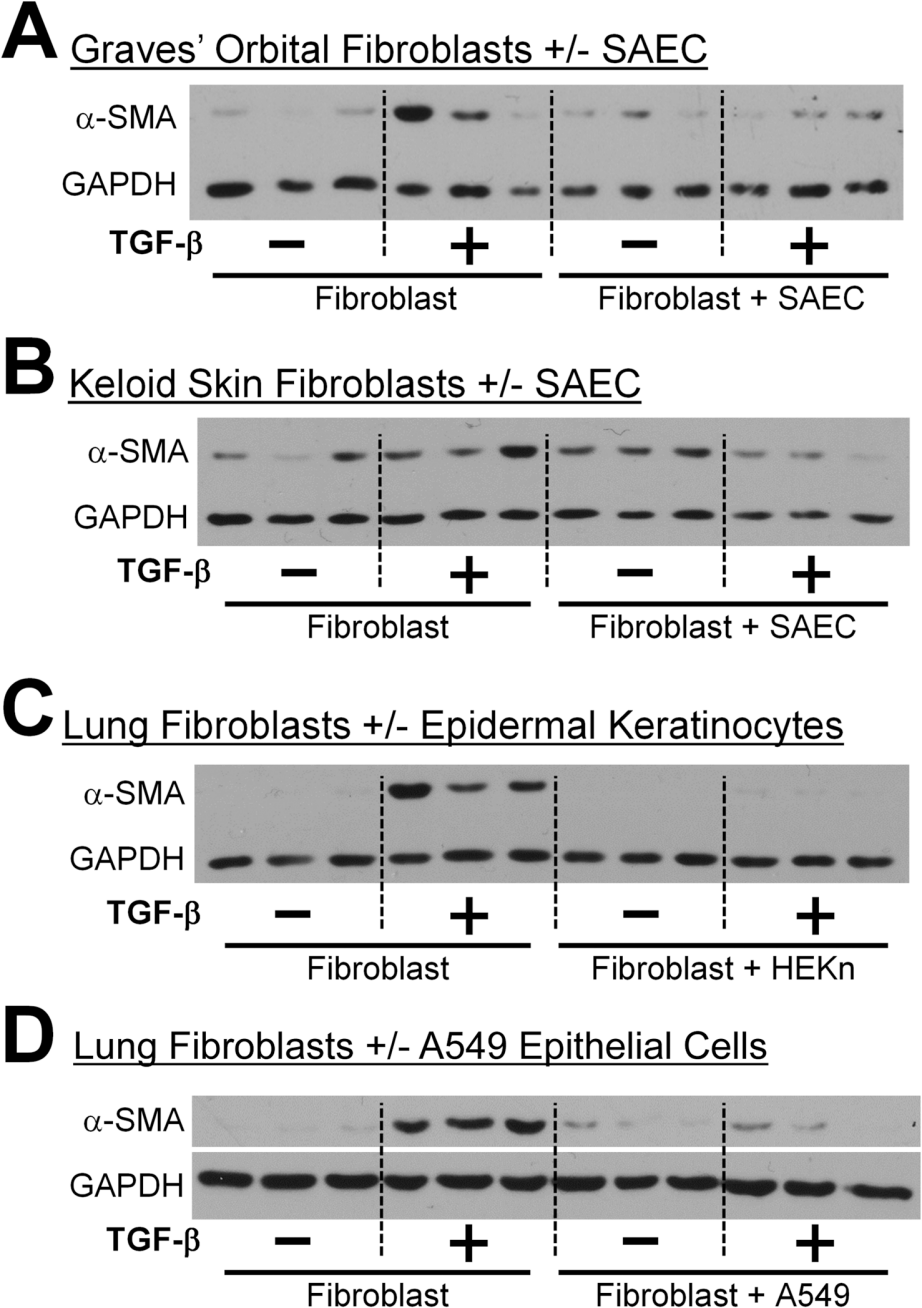
Epithelial cells inhibit TGF-β induced α-SMA protein expression in fibroblasts irrespective of their tissue origin. Multiple strains of human epithelial cells and fibroblasts were treated with or without 5ng/ml TGF-β and co-cultured for 72 hours. (A) SAECs co-cultured with Graves’ orbital fibroblasts. (B) SAECs co-cultured with keloid fibroblasts. (C) Human epidermal keratinocytes-neonatal co-cultured with HLFs. (D) A549 cells co-cultured with HLFs. α-SMA protein expression was analyzed by western blot. Each lane represents a replicate culture. Representative blots are shown from at least two independent experiments per condition.

Epithelial cells and fibroblasts are contiguous in many injury and wound healing situations. Given our results (Figs 1 and 2) that primary lung epithelial cells are capable of inhibiting TGF-β induced α-SMA expression in both normal and fibrotic lung fibroblasts, we examined whether epithelial cells were capable of protecting fibroblasts from pro-scarring insult irrespective of their tissue origin. Graves’ ophthalmopathy is an autoimmune disease in which orbital fibroblasts proliferate and differentiate to myofibroblasts in response to infiltrating T cells [29, 33]. Keloid scars represent a fibroproliferative disorder characterized by abnormal wound healing and high levels of α-SMA and collagen expression [34]. Using the same co-culture system, we cultured Graves’ orbital and keloid fibroblasts with SAECs. Interestingly, SAECs inhibited TGF-β stimulated α-SMA expression in both Graves’ orbital fibroblasts and keloid fibroblasts. (Fig 3A and 3B).

Next, we also wanted to determine whether epithelial cells from other sources could inhibit myofibroblast differentiation of lung fibroblasts. We co-cultured lung fibroblasts with either primary human neonatal epidermal keratinocytes (HEKn) or the human lung alveolar epithelial cell line, A549, in the presence of TGF-β. Epithelial cells from diverse origins suppressed myofibroblast differentiation (Fig 3C and 3D).

### Human lung epithelial cells decrease collagen gel contraction and migration of human lung fibroblasts

**Fig 4:**
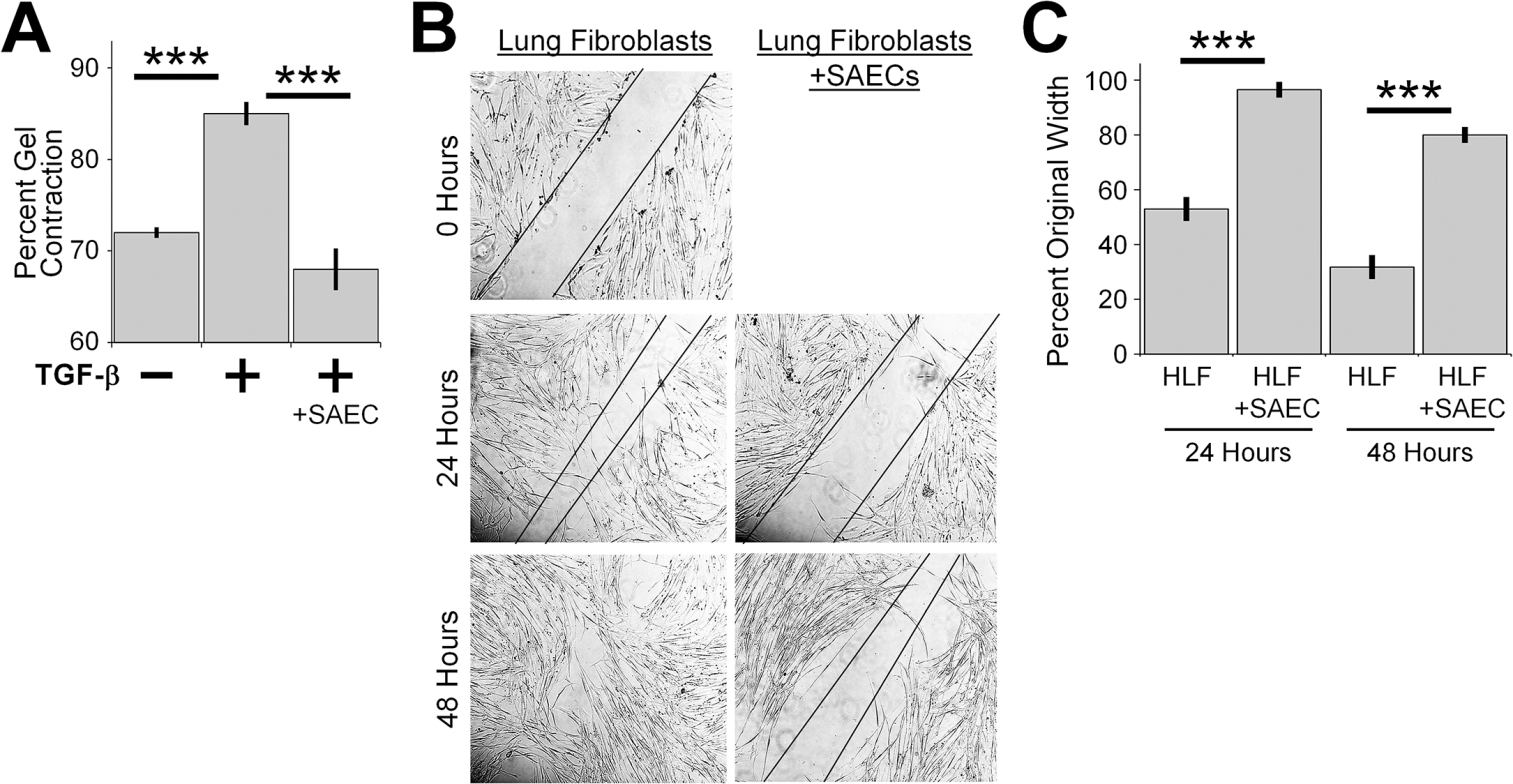
SAECs inhibit collagen gel contraction and fibroblast migration. (A) HLFs were seeded into collagen gels and floated in medium containing 5ng/ml TGF-β. SAECs were grown separately on Transwell inserts and added to the wells. After 72 hours gels were weighed and percent contraction was calculated. n = 3–4 per group. (B) HLFs were grown on 6-well plates until they formed a confluent monolayer. A scratch wound was made on HLF monolayer, cells were washed with PBS and co-cultured with SAECs. HLF migration was tracked over time and imaged by phase contrast microscopy. (C) The scratch assay was performed on 3 replicate cultures for each condition. Each culture was photographed at 3 locations, and the width of the scratch was determined at 3 positions in each photograph (total of 9 measurements per condition), and percentage of original width was calculated by measuring the width between the edges of the scratch wound in three distinctive areas of each image. Data shown are mean ± SEM. *** = p<0.001 by ANOVA.

We also studied whether epithelial cells were capable of inhibiting other key pro-fibrotic effector functions exhibited by lung fibroblasts, specifically increased contractile ability and migration. Collagen gel contraction assays measure the ability of fibroblasts to organize and contract a collagen matrix *in vitro*. As predicted, TGF-β increased contraction of collagen gels by the fibroblasts, but the fibroblasts that were co-cultured with SAECs failed to contract in response to TGF-β (Fig 4A).

Next, we evaluated whether epithelial cells were capable of inhibiting fibroblast migration. Fibroblasts that were co-cultured with SAECs showed a reduction in migration capability at 24 and 48 hours (Fig 4B and 4C). These data indicate that normal lung epithelial cells in the presence of HLFs are capable of inhibiting properties normally associated with a fibrotic phenotype.

### Epithelial cells inhibit TGF-β induced fibroblast proliferation without affecting cell viability

**Fig 5:**
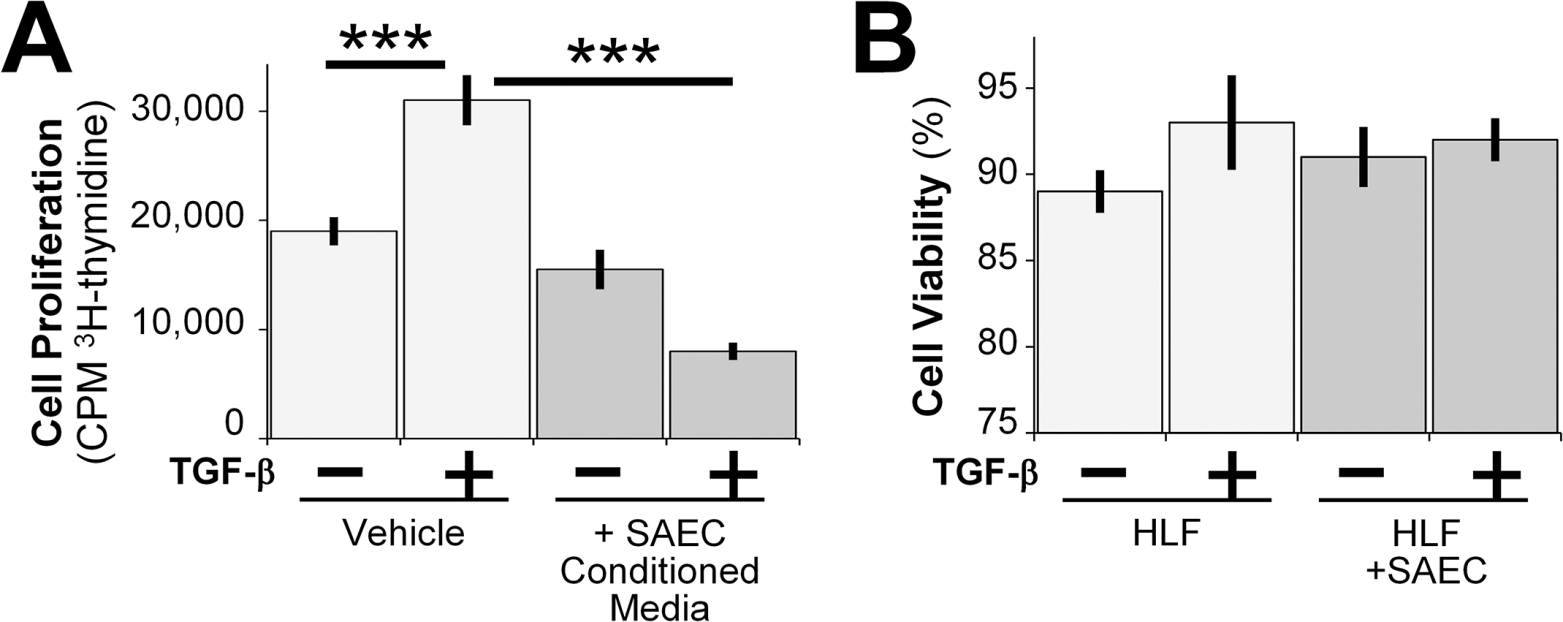
SAECs inhibit TGF-β induced fibroblast proliferation without affecting cell viability. (A) HLFs were treated with 5ng/ml TGF-β in fresh SAGM or in SAEC conditioned medium for 24 hours. Cells were allowed to incorporate ^3^H-thymidine for another 18 hours. n = 6 per group. Data shown are mean ± SD. *** = p<0.001 by ANOVA. (B) HLFs were treated with 5ng/ml TGF-β in the presence or absence SAECs for 72 hours and HLF viability was measured by trypan blue dye exclusion method. n = 3 per group.

Another important phenotypic characteristic of fibroblasts from IPF patients is proliferation [35, 36]. To investigate whether epithelial cells were capable of inhibiting TGF-β induced fibroblast proliferation, we examined cell proliferation using the ^3^H-thymidine incorporation assay. Because we could not perform Transwell-style co-cultures in the 96-well format, we treated HLFs with or without TGF-β in SAEC conditioned medium. Epithelial cell conditioned medium significantly reduced the TGF-β induced fibroblast proliferation without affecting the baseline untreated cell proliferation rate (Fig 5A). We also assessed cell viability of HLFs that were co-cultured with SAECs for 72 hours using trypan blue dye exclusion. No significant differences in viability were noted among the fibroblast alone versus co-culture groups (Fig 5B).

### SAECs exert anti-fibrotic effects through the soluble mediator PGE_2_

**Fig 6:**
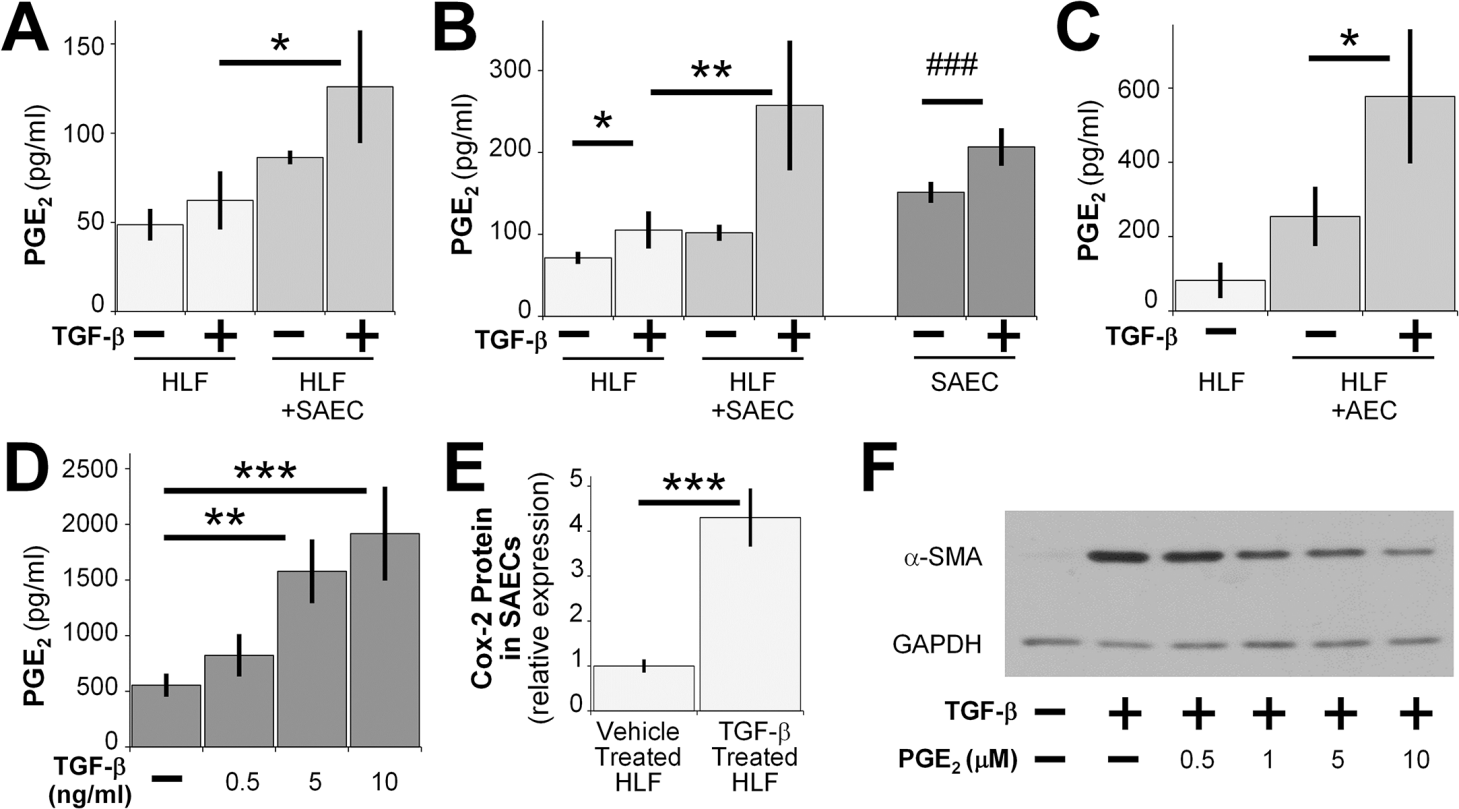
SAECs exert anti-fibrotic effects through the soluble mediator PGE_2_. PGE_2_ in culture medium from SAEC-HLF co-cultures was measured by competitive EIA at 24 hours (A) and 72 hours (B) after TGF-β treatment. (C) PGE_2_ concentrations in culture medium from AEC-HLF co-cultures were measured at 72 hours. (D) PGE_2_ in culture medium from SAECs that were treated with TGF-β was measured at 48 hours post treatment. Data shown are mean ± SEM for n = 3 replicates. *** = p<0.001, ** = p<0.01 and * = p<0.05 by ANOVA. ### = p<0.001 by student’s t-test. (E) COX-2 protein expression was analyzed by western blot in SAECs that were co-cultured with HLFs and treated with or without 5ng/ml TGF-β. Densitometry of n = 3 replicates, normalized to untreated control. Data shown are mean ± SD. *** = p<0.001 by student’s t-test. (F) HLFs were treated with or without 5ng/ml TGF-β, or with 5ng/ml TGF-β and increasing concentrations of exogenous PGE_2_ for 24 hours in serum-free MEM. α-SMA protein expression was analyzed by western blot. A representative blot is shown.

Because the co-culture system used in our current study prevents direct cell-to-cell contact, we hypothesized that paracrine mediators produced by the SAECs were responsible for the inhibitory effect described above. One candidate for soluble antifibrotic mediator is prostaglandin E_2_ (PGE_2_). Exogenous PGE_2_ has been shown to have diverse anti-fibrotic effects on mouse lung fibroblasts, as well as human lung fibroblasts [19, 20, 37]. To determine whether lung epithelial cells secrete PGE_2_, we measured PGE_2_ levels in cell culture medium from the fibroblast compartment of fibroblast-SAEC co-cultures, fibroblast-primary alveolar epithelial cell co-cultures and SAEC single cultures at different time points using a competitive enzyme immunoassay (EIA). Cultures of SAEC alone had higher levels of PGE_2_ at 72 hours, and this was further increased by TGF-β treatment (Fig 6B). Cultures of fibroblasts alone had less PGE_2_ compared to cultures of SAECs alone and TGF-β treatment did not significantly increase PGE_2_ levels in the culture medium. Increased levels of PGE_2_ were detected in the medium of fibroblasts that had been co-cultured with SAECs at 24 and 72 hours (Fig 6A and 6B). Similar results were seen in primary alveolar epithelial cell (AEC)-HLF co-culture at 72 hours post co-culture (Fig 6C). Furthermore, SAECs treated with increasing doses of TGF-β showed a dose dependent increase of PGE_2_ levels in cell culture medium (Fig 6D).

Cyclooxygenase (COX) 2 is the rate-limiting enzyme, in the conversion of arachidonic acid to prostaglandins including PGE_2_. Therefore, we investigated COX-2 expression in SAECs that were co-cultured with HLFs. COX-2 protein expression was higher in epithelial cells that were co-cultured with HLFs treated with TGF-β than in epithelial cells co-cultured with HLFs not treated with TGF-β (Fig 6E).

We next examined whether exogenous PGE_2_ was capable of inhibiting TGF-β induced α-SMA expression in primary adult HLFs. HLFs treated with PGE_2_ showed a dose-dependent reduction of TGF-β induced α-SMA protein expression (Fig 6F). Taken together, these data suggest that normal SAECs are capable of inhibiting TGF-β induced α-SMA protein expression through the potent anti-fibrotic mediator PGE_2_.

**Fig 7:**
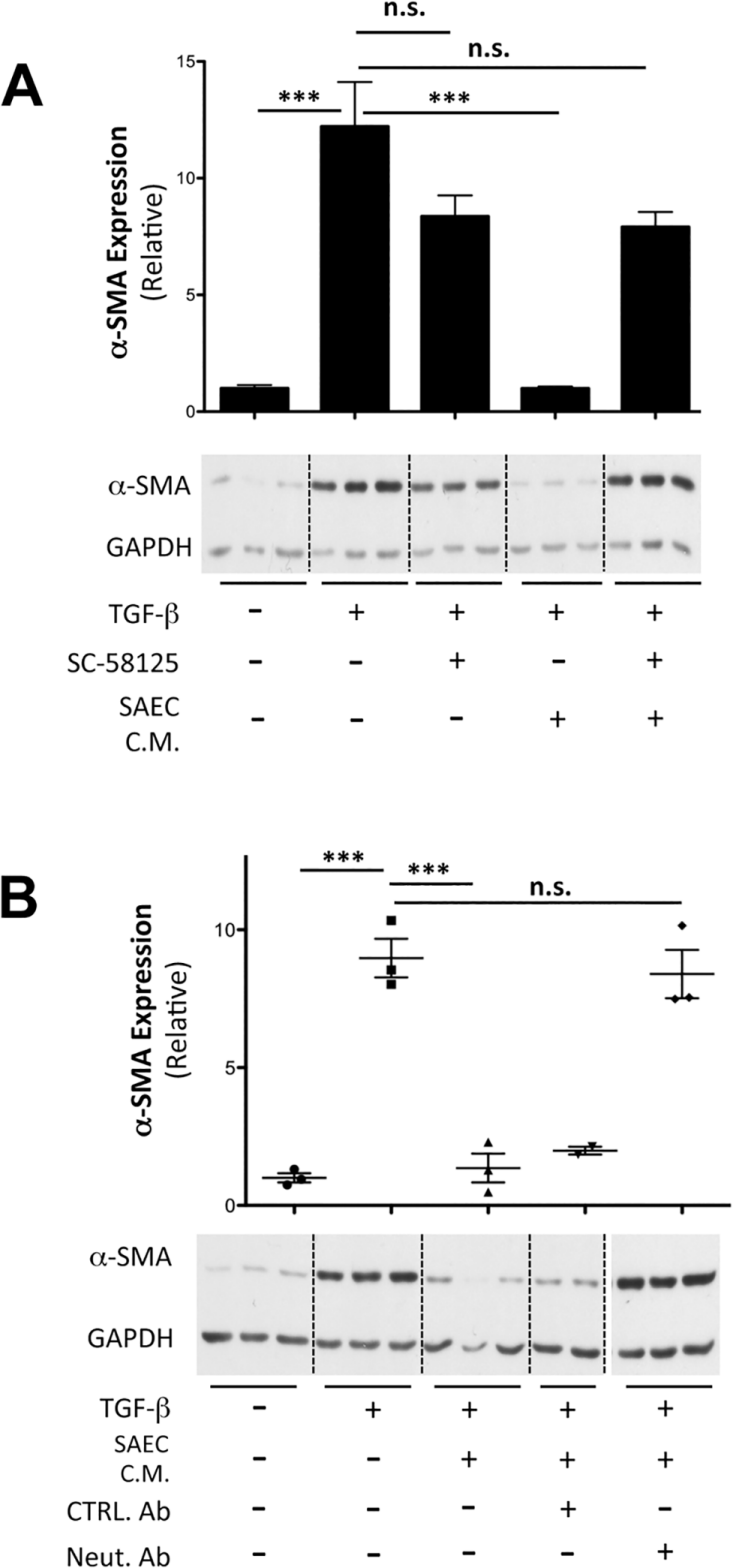
Epithelial cell PGE_2_ production is COX-2 dependent and is reversed by a PGE_2_ neutralizing antibody. (A) Conditioned medium was collected from SAECs treated with or without 70nM SC-58125 for 24 hours. HLFs were treated with 5ng/ml TGF-β either in the presence or absence of SC58125 alone, or in the presence of SAEC conditioned medium from control or SC58125-treated SAECs. α−SMA protein expression was analyzed after 48 hours by western blot and densitometric analysis. (B) Conditioned medium from SAECs was neutralized by addition of 10μg/ml of anti- PGE_2_ antibody, 2B5. HLFs were treated with 5 ng/ml TGF-β in either fresh SAGM, SAEC conditioned medium, or PGE_2_ neutralized SAEC conditioned medium for 48 hours. α-SMA protein expression was analyzed by western blot. A representative blot from multiple independent experiments is shown. Samples were resolved on the same gel, and intervening irrelevant lanes are not shown. Note that, Neut. Ab– 2B5, PGE_2_ neutralizing antibody, CTRL Ab–control antibody, n.s.–not significant. Data shown are mean ± SEM for n = 3 replicates. *** = p<0.001 by ANOVA.

We used two approaches to confirm that SAEC-derived PGE_2_ is responsible for inhibiting myofibroblast differentiation. First, we incubated primary HLFs treated with TGF-β with either the COX-2 inhibitor SC58125, or with conditioned medium from SAECs that had been treated with SC58125 [38]. SC58125 alone did not increase myofibroblast differentiation in TGF-β-stimulated HLFs, demonstrating that fibroblast autologous COX-2 activity does not retard differentiation (Fig 7A). However, conditioned medium from untreated SAECs, but not from SAECs treated with SC58215, was able to inhibit α-SMA expression. This confirms that the antifibrotic effector is a soluble molecule downstream of COX-2, and is produced by SAECs and not HLFs. To further identify that the active product was PGE_2_, we incubated SAEC-conditioned medium with anti-PGE_2_ neutralizing antibody [30, 39, 40] before applying to the HLFs. HLFs treated with SAEC-conditioned medium in the presence of TGF-β had reduced α-SMA protein expression levels. Pre-treating SAEC-conditioned medium with anti-PGE_2_ neutralizing antibody significantly reversed the inhibitory effect exerted by SAEC-conditioned medium (Fig 7B).

## Discussion

Herein, we provide the first direct experimental evidence that healthy lung epithelial cells can exert a wide array of anti-fibrotic effects on lung fibroblasts, even in the presence of the strong pro-fibrotic stimulus TGF-β. Furthermore, we show that epithelial cells from different tissue origins can inhibit TGF-β stimulated myofibroblast differentiation of fibroblasts from multiple sites. This suggests that epithelial-fibroblast cross-talk is a generalized phenomenon that occurs widely, is important for maintaining normal tissue homeostasis, and may represent a new therapeutic target in multiple scarring diseases.

The role of the lung epithelium in IPF remains controversial, and is under active investigation. However, a number of reports show IPF lung epithelium exhibit changes, such as hyperplastic type 2 alveolar epithelial cells, that co-express markers of other epithelial cells, microscopic epithelial denudation, and honeycomb lesions comprised of bronchiolar epithelium [8]. Two studies in mice showed that targeted injury to lung epithelial cells induces lung fibrosis [13, 41]. Here, rather than consider how a damaged epithelium might promote fibrosis, we investigated how healthy lung epithelium acts to maintain fibroblast homeostasis against the pro-fibrotic insult, TGF-β. We show strong evidence that primary human small airway epithelial cells are capable of inhibiting a diverse array of pro-fibrotic functions (Figs 1, 4 and 5). This evidence is strengthened by the fact that we used primary human cell strains of epithelial cells and fibroblasts from different donors (Figs 1 and 2). To evaluate the generality of this phenomenon, we performed co-culture experiments using fibroblasts and epithelial cells from non-matching tissue types. In all cases the epithelial cells were capable of potently inhibiting α SMA expression in the fibroblasts with which they were “cross co-cultured” (Fig 3). Interestingly, others have reported epithelial cell destruction in keloid scars [42], similar to reports showing loss of epithelial cells in lung fibrosis [43]. Our new results highlight the existence of an epithelial-mesenchymal interaction, independent of tissue type, that is important in maintaining tissue homeostasis and normal wound healing response.

Epithelial-mesenchymal crosstalk could be mediated through soluble factors that act either in an autocrine or a paracrine manner or by cell adhesion complexes, which require direct cell-cell contact. The Transwell co-culture model limits direct cell-cell contact, but allows small molecules to act as messengers between the two compartments. Our results demonstrate that SAECs produce more PGE_2_ in response to TGF-β when cultured alone, as well as in co-culture, compared to HLFs (Fig 6). This is the first report showing that lung epithelial cells upregulate PGE_2_ and COX-2 in response to TGF-β stimulation (Fig 6). This could be as a result of epithelial cells attempting to maintain tissue homeostasis in response to a strong fibrotic stimulus. We used an irreversible COX-2 inhibitor and a PGE_2_ neutralizing antibody to demonstrate that the inhibitory properties of SAEC co-culture are conveyed largely through PGE_2_ (Fig 7). It is worth noting that the level of PGE_2_ measured in cell culture supernatants is lower than the concentration of exogenous PGE_2_ required for inhibition of TGF-β induced α-SMA expression. The steady-state level of PGE_2_ measured in co-culture represents an equilibrium between production by SAECs and uptake by SAECs and fibroblasts, and probably reflects a low continual synthesis that maintains its biological effect. In contrast, a single bolus of exogenous PGE_2_ added at the beginning of the experiment must be large enough to account for degradation and inactivation in culture over time, so it is not surprising that the effective concentration for a single bolus of PGE_2_ is higher than the levels in co-culture.

It has been previously reported that addition of exogenous PGE_2_ into cell culture medium inhibits pro-fibrotic effector functions of human lung fibroblasts *in vitro* [19–21, 44]. It was also reported that mouse and rat epithelial cells could inhibit serum-stimulated proliferation of lung fibroblasts via PGE_2_ [16, 17], but other pro-fibrotic effector functions were not measured, and a pro-fibrotic stimulus was not tested. Here, we report that healthy lung epithelial cells are an important source of PGE_2_, that normal human lung epithelial cells upregulate PGE_2_ in response to TGF-β, and that human lung epithelial cells inhibit myofibroblast differentiation even in the presence of TGF-β. Our results are consistent with data that IPF patients have reduced COX-2 expression and PGE_2_ production in their lungs [15, 45–47], and in bronchial epithelial cells [48]. Deficiency in microsomal prostaglandin E synthase-1 (mPGES-1), the enzyme that converts prostaglandin H_2_ (PGH_2_) to PGE_2_ worsens bleomycin induced pulmonary fibrosis [49]. PGE_2_ has also being shown to be effective in protecting murine lungs against bleomycin induced pulmonary fibrosis [50, 51].

We previously reported that IPF fibroblasts are resistant to some anti-fibrotic treatments *in vitro* [52], and it has been shown that fibroblasts derived from patients with IPF fail to upregulate COX-2 and PGE_2_ synthesis when stimulated with LPS or IL-1 [45]. Moreover, multiple studies showed that IPF fibroblasts are less responsive to exogenous PGE_2_ [53, 54]. Interestingly, SAECs were equally effective in inhibiting TGF-β induced α−SMA expression in normal and IPF fibroblasts. While PGE_2_ appears to convey the majority of the antifibrotic effects of SAECs, we cannot rule out that other mediators might also contribute. If present, these additional mediators might modify IPF fibroblast responses to PGE_2_, explaining why IPF fibroblasts are resistant to exogenous PGE_2_ but not SAECs. Whether or not PGE_2_ is the sole antifibrotic mediator, our results suggest that the underlying cross-talk between epithelial cells and fibroblasts is intact in IPF and might be amenable/accessible to new therapeutic modification.

Fibroblasts are also reported to be a source of PGE_2_ in some reports [55–57], suggesting that fibroblasts can dampen their own myofibroblast differentiation via an autocrine pathway. However, in our experiments, TGF-β did not upregulate PGE_2_ production in fibroblasts cultured alone, and blockade of COX-2 with SC58125 did not result in increased myofibroblast differentiation. Therefore, we conclude that autocrine production of PGE_2_ by lung fibroblasts does not significantly contribute to the results we observed.

Lung alveolar epithelium consists of type I and type II cells. IPF is characterized in part by the loss of type I cells that are important for gas exchange and the accumulation of hyperplastic type II cells and bronchiolar cuboidal cells [58]. Therefore, it would be intriguing to consider whether type I or type II cell were more effective at inhibiting myofibroblast differentiation. The majority of studies evaluating alveolar epithelial prostaglandin production used type II AECs [17, 59], and to our knowledge there are no current reports comparing PGE_2_ production between two cell types. Here, we show that freshly isolated primary alveolar epithelial cells cultured under conditions that favor the type I phenotype [22] produced high levels of PGE_2_ (Fig 6D) and were potent inhibitors of myofibroblast differentiation (Fig 1D). A549 cells, an alveolar type II-like cell line derived from an adenocarcinoma, were also effective inhibitors of myofibroblast differentiation (Fig 3D), with no obvious advantages or disadvantages over the freshly isolated AECs. However, A549 cells may not accurately represent all the phenotypes of type II cells, and we were unable to rigorously investigate the effects of type I and type II cells due to limited availability of donor tissue. However, it is intriguing that type I cells are strongly protective, given the relative paucity of type I cells in fibrotic lungs [58].

Taken together, our data suggests that normal healthy lung epithelium is indispensible for maintaining tissue homeostasis. Epithelial injury or damage leading to lung fibrosis could cause reduction of epithelial cell number and/or reduce COX-2 and PGE_2_ production resulting in a loss of the protective antifibrotic activity of the epithelium. In turn, this would trigger an amplified fibrotic response, leading to tissue fibrosis. In future studies, we hope to further explore the antifibrotic properties of the epithelium using known risk factors of IPF as injury stimuli to the epithelium, as well as to further dissect other potentially important pathways. However, it is noteworthy that most of the known lung injury stimuli such as TGF-β (Fig 6), and silica [60] causes an upregulation of PGE_2_ production in lung epithelial cells, yet there is diminished PGE_2_ in IPF patients. This suggests that loss of epithelial cells and thereby loss of protective effects might be a crucial step in amplifying the pathogenic cascade in fibrosis. A recent publication supports this notion by showing that transplantation of type II alveolar epithelial cells reverses bleomycin-induced lung fibrosis [61].

Our findings reinforces PGE_2_ as a viable therapeutic option for organ fibrosis and also opens up potential treatment options based on restoring dysfunctional epithelium in IPF lungs or at least restoring the important signaling mechanisms that maintains the tissue homeostasis. Our data encourages these options, and hope for clinical feasibility, as our co-culture model was equally effective in fibrotic and normal lung fibroblasts.

## Supporting Information

S1 Dataset In accordance with the PLOS ONE policy on data availability, the raw data for the figures included in this report are located in an online data supplement S1 dataset. (XLSX)
